# Estimation of COVID-19 vaccine effectiveness against infections and severe outcomes using routine surveillance data in Kosovo, July—September 2021

**DOI:** 10.1371/journal.pone.0305629

**Published:** 2024-07-24

**Authors:** Albiona Rashiti-Bytyçi, Emily White Johansson, Pranvera Kaçaniku-Gunga, Kostas Danis, Anja Schoeps, Achim Dörre, Fetije Fetaj, Arijana Kalaveshi

**Affiliations:** 1 National Institute of Public Health, (NIPHK), Prishtina, Kosovo; 2 The Mediterranean and Black Sea Programme for Intervention Epidemiology Training (MediPIET), European Centre for Disease Prevention and Control (ECDC), Stockholm, Sweden; 3 European Centre for Disease Prevention and Control (ECDC), Stockholm, Sweden; 4 Landesuntersuchungsamt Rheinland-Pfalz, Koblenz, Germany; 5 European Programme for Intervention Epidemiology Training (EPIET), European Centre for Disease Prevention and Control (ECDC), Stockholm, Sweden; 6 Postgraduate Training for Applied Epidemiology (PAE), Robert Koch Institute (RKI), Berlin, Germany; 7 Department of Infectious Disease Epidemiology, Robert Koch Institute, Berlin, Germany; University of Business and Technology, ALBANIA

## Abstract

**Background:**

COVID-19 vaccines have proven effective against severe outcomes in many settings, yet vaccine effectiveness (VE) estimates remain lacking for Kosovo. We aimed to estimate VE against COVID-19 infections, hospitalisations, and deaths for one and two vaccine doses during the fourth pandemic wave in July—September 2021, the period when vaccination initially became widely available.

**Methods:**

We analysed routine surveillance data to define cases and vaccination status as partially (one dose) or completely (two doses) vaccinated. We used the screening method to calculate the proportion of cases with the outcomes vaccinated (PCV). The proportion of the population vaccinated (PPV) was based on numbers vaccinated and the Kosovo population estimate on 30/09/2021.

**Results:**

Between July—September 2021, 51,804 COVID-19 cases were reported in Kosovo with 9.3% of cases partially and 3.4% completely vaccinated. Estimated vaccine effectiveness for one dose was 93.1% (95%CI:92.9–93.2%) for infections, 90.3% (95%CI:88.8–91.7%) for hospitalisations, and 90.3% (95%CI:88.4–92.1%) for deaths. Estimated vaccine effectiveness for two doses was 97.8% (95%CI:97.6–97.9%) for infections, 94.5% (95%CI:93.3–95.6%) for hospitalisations, and 94.2% (95%CI: 93.7–96.5%) for deaths.

**Conclusions:**

This study provides real-world evidence for COVID-19 vaccine effectiveness in Kosovo using routine administrative data sources and the screening method. COVID-19 vaccine effectiveness against infections and severe outcomes in Kosovo was higher with two vaccine doses than one dose, which is in accordance with findings from other study designs and settings. Using the screening method in our study reflects an important initial methodology for estimating vaccine effectiveness with routine surveillance that may be particularly important for low- and middle-income settings with less robust surveillance systems or fewer opportunities to conduct more robust vaccine effectiveness study designs.

## Introduction

The COVID-19 pandemic has been a global challenge resulting in about six hundred million confirmed cases and over six million confirmed deaths globally [1]. In Kosovo, according to the data of the National Institute of Public Health in Kosovo (NIPHK), the COVID-19 pandemic waves from 2020 to 2022 resulted in the cumulative number of 272,258 reported cases. As of March 13, 2020, a total of 3,202 deaths attributable to COVID-19 have been recorded with a case-fatality of 1.17% [2].

Safe and effective COVID-19 vaccines are a powerful tool for ensuring public health and controlling the pandemic. Results from observational studies carried out to date have indicated that the vaccines authorized in the EU/EEA are highly protective against severe COVID-19, hospitalization, and death [3].

In Kosovo, vaccination against COVID-19 started on March 2021 based on the State Plan for vaccination against COVID-19, drawn up by the Committee for immunization against COVID-19 within the Ministry of Health. Additionally, as vaccine supply was constrained, most countries opted to prioritize vaccination in high-risk populations [4]. During the initial roll out, vaccine prioritization focused on health workers; residents in nursing homes and all social workers; people aged over 80 years; as well as people with chronic diseases (people on dialysis, people with diabetes, cardiovascular disease). The second phase included the following prioritization: people aged 65–79 years; other persons with chronic diseases; teachers and security forces involved in the management of COVID-19. The third phase included vaccination of approximately 50% of the general population, including the age group 40–64 years, the remaining population with significant health conditions, and public sector workers [5]. Despite the widespread roll out of COVID-19 vaccination to the population, there remains a lack of evidence for vaccine effectiveness for the Kosovo population.

Therefore, the aim of this study was to estimate COVID-19 vaccine effectiveness against COVID-19 infections and severe outcomes, including hospitalization and death, during the fourth pandemic wave in Kosovo from July 26, 2021, to September 26, 2021.

## Methods

### Study design and data sources

The study was conducted in Kosovo from July 26, 2021 to September 26, 2021 during the fourth pandemic using routine surveillance data and the screening method [6].

For the study, we used data from the COVID-19 surveillance system, the immunization registry against COVID-19 and the population registry of Kosovo. COVID-19 cases were tested with PCR (Polymerase chain reaction) and RAT (Rapid antigen tests) in public and private laboratories and were reported daily to the Emergency Operations Center at National Institute of Public Health in Kosovo. COVID-19 surveillance system collected data on positive COVID-19 cases, including age, gender, place of residence, symptoms, number of contacts, hospitalizations, and deaths. Also, after the start of the application of vaccines against COVID-19, the vaccination date and number of doses were reported incompletely into the surveillance system. The PCV (proportion of cases vaccinated) was calculated using data from the COVID-19 surveillance system. The PPV (proportion of population vaccinated) was calculated using data from the vaccination registration as well as the total population from the Statistics Agency’s register.

### Definitions

Since vaccination began during pandemic wave 3 and 4, COVID-19 vaccination with one dose was considered partially immunized and COVID-19 vaccination with two doses was considered completely vaccinated. At that time, no one in the population had received three or more doses. A confirmed COVID-19 case was defined as a patient with a reported COVID-19 positive test result using PCR or RAT between July 26 and September 26, 2021 (fourth wave).

### Data analysis

Popularized by the landmark publication in 1993 [5] and first used in 1980 [7], the screening method [6] is used to estimate vaccine effectiveness using routine surveillance data. This method requires availability of two types of information: (i) the proportion of vaccinated individuals in the population of interest and (ii) the proportion of vaccinated among cases. However, no vaccination information is needed for those who are not infected. This makes the screening method a particularly suitable method for conducting vaccine effectiveness studies in low resource settings, as information about reported cases is much easier to obtain in routine surveillance.

According to Farrington [6], the formula for calculating vaccine effectiveness (VE) by the screening method is:

VE=1−PCV/(1−PCV)×(1−PPV)/PPV

The proportion of cases vaccinated (PCV) was derived from the COVID-19 surveillance system. We performed data cleaning before analyses, excluding any probable double instances. To calculate PCV, the denominator included all COVID-19 cases of all age groups for the time period reported in the surveillance system who were hospitalized, or died, while the numerator included only vaccinated cases among those in the denominator. To calculate PPV, the denominator included the total census population estimate on September 30, 2021 in Kosovo, while the numerator was the number vaccinated among this population. We calculated confidence intervals (CI) for the vaccine effectiveness (VE) estimate based on a binomial model with normal approximation for infections, hospitalized cases and deaths, for a single dose and for two doses.

## Results

**Fig 1:**
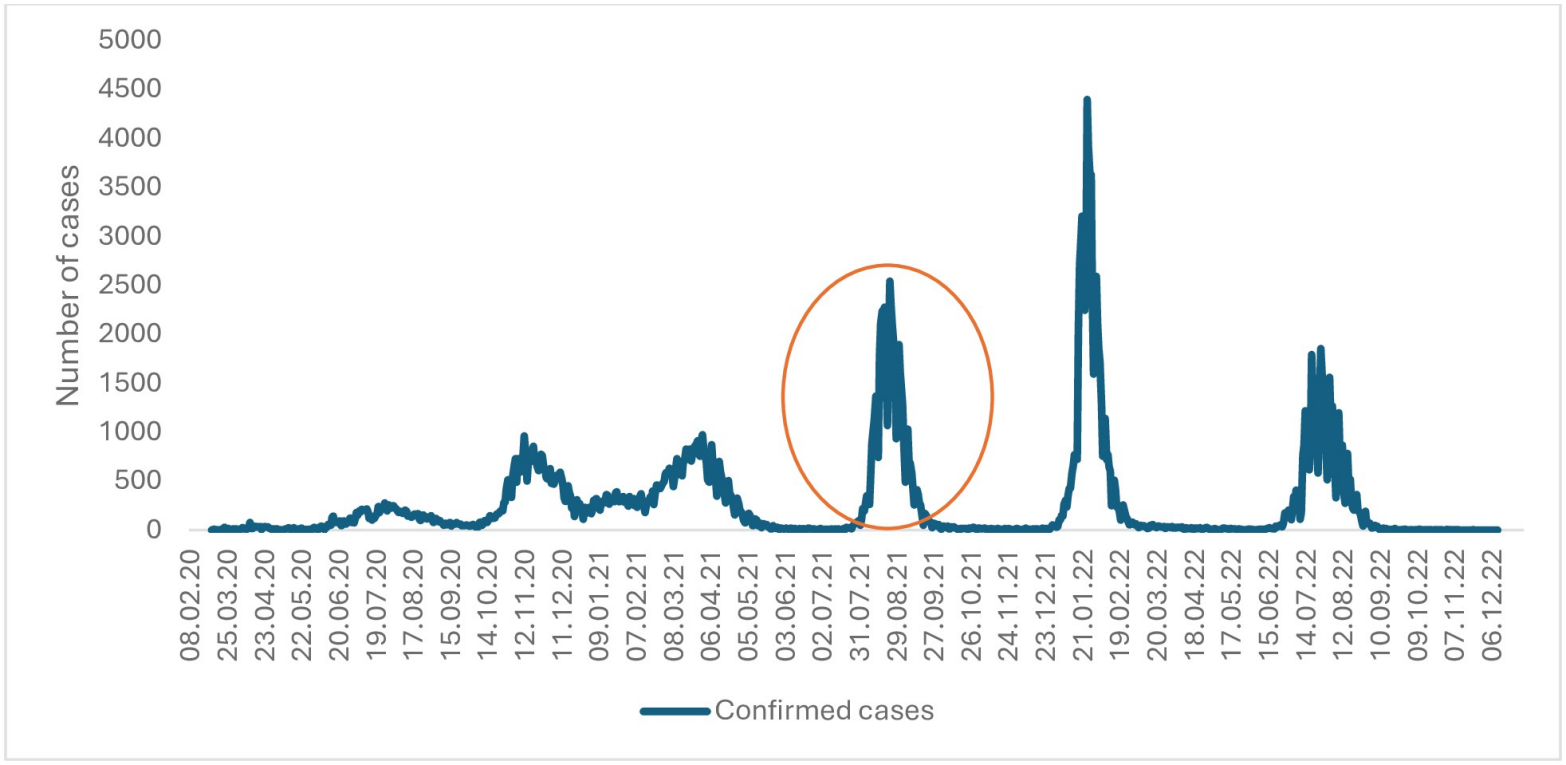
COVID-19 confirmed cases in Kosovo, 2020–2022 (circled—The fourth COVID-19 pandemic wave—Study period).

During the fourth COVID-19 pandemic in Kosovo from July 26, 2021, to September 26, 2021, 51,804 confirmed COVID-19 cases were recorded in the national surveillance system among the Kosovo population (Fig 1).

Among these confirmed cases, 56% were female; 80% were reported in the adult population (19–64 years). Among confirmed COVID-19 cases, 9% were vaccinated with one dose (partially vaccinated), 3% with two doses (completely vaccinated), while 84% of confirmed COVID-19 cases were unvaccinated (Table 1). 2.3% of cases (1,194) were hospitalized, and 1.4% (701) died.

**Table 1:** Characteristics of Covid-19 confirmed cases during the fourth pandemic wave in Kosovo, July 26, 2021 to September 26, 2021.

Characteristics		Covid-19 cases n (%)
Sex	Male	22,849 (44.1)
	Female	28,955 (55.8)
Age groups	0–18	5,465 (10.5)
	19–64	41,182 (79.5)
	65–79	4,363 (8.4)
	80+	7,94 (1.5)
Number of Covid-19 vaccine doses administered	None	43,507 (83.9)
	One dose	48,31 (9.3)
	Two doses	1,747 (3.4)
	Three doses	0 (0.0)
	Not reported	1,719 (3.3)

Based on the screening method, the vaccine effectiveness of having one COVID-19 vaccine dose during the study period was 93.1% (95%CI, 92.9–93.2%) for infections, 90.3% (95%CI, 88.8–91.7%) for hospitalizations and 90.3% (95%CI, 88.4–92.1%) for deaths (Table 2), indicating a 93.1%, 90.3%, and 90.3% reduction in the risk of developing the outcome of COVID-19 infection, hospitalization, or death, respectively, among people receiving one vaccine dose compared with unvaccinated individuals.

**Table 2:** Estimates for COVID-19 vaccine effectiveness against infections, hospitalizations, and deaths, by one and two doses, 4^th^ wave of pandemic in Kosovo, July 26, 2021, to September 26, 2021.

Study outcome	Partially vaccinated			Completely vaccinated		
	Vaccinated/Total	VE (%)	95% CI	Vaccinated/Total	VE (%)	95% CI
**Infections**	6883/51804	**93.1**	92.9–93.2	1747/51804	**97.8**	97.6–97.8
**Hospitalizations**	212/1194	**90.3**	88.8–91.7	95/1194	**94.5**	93.3–95.6
**Deaths**	129/701	**90.3**	88.4–92.1	49/701	**95.2**	93.7–96.5

*Estimation of vaccination protection based on the screening method [6]

For vaccination with two COVID-19 doses, the vaccine effectiveness estimates were 97.8% (95%CI, 97.6–97.8%) for infections, 94.5% (95%CI, 93.3–95.6%) for hospitalizations and 94.2% (95%CI, 93.7–96.5%) for death, indicating a 97.8%, 94.5%, and 94.2% reduction in the risk of developing the outcome of COVID-19 infection, hospitalization, or death, respectively, among people receiving two vaccine doses compared with unvaccinated individuals.

## Discussion

Our study provides real-world evidence using routine administrative data sources to estimate COVID-19 vaccine effectiveness for Kosovo during the fourth pandemic wave, when the Delta variant and later Delta plus variant were dominant. COVID-19 vaccine effectiveness against infection and severe outcome in Kosovo was above 90% even with one vaccine dose. In addition, the difference in vaccine effectiveness against infection, hospitalization, and death with two doses of vaccine as compared to one dose was approximately 3 to 4 percentage points higher.

This study was restricted to the fourth pandemic wave since vaccination was first introduced widely in the population at this time and surveillance data did not include information on time since vaccination. This study period restriction therefore ensures that waning immunity would not affect results. During that period, vaccination was widely available, and most people were vaccinated. At the same time, the fourth COVID-19 pandemic wave in Kosovo was also the period with the highest number of deaths with a total of 701 deaths (case-fatality 1.35%). This wave is characterized by the large distribution of Delta and Delta Plus variants of SARS-CoV-2.

Findings of the study are in accordance with evidence from several published studies using cohort study designs from other settings [8–14]. Similar to our results, these studies reported an overall vaccine effectiveness against symptomatic SARS-CoV-2 infection, of 73% (95% CI: 62–81%) for >14 days post-second vaccine dose [8]. Other studies reported that vaccines successfully lowered the risk of SARS-CoV-2 infection at the community level [9]. The cohort study [10] indicated a vaccine effectiveness (VE) of 91.8–94.9% against COVID-19 infections as they transitioned from unvaccinated to fully vaccinated status. Other observational studies reported high or very high effectiveness of five different vaccines in the prevention SARS-CoV-2 infection and COVID-19-related death [11]. Another study [12] indicated the additional benefit of a second booster dose in terms of SARS-CoV-2 infection and COVID-19-related mortality. Several other studies suggested that COVID-19 vaccinations prevent SARS-CoV-2 infections and decrease the number of COVID-19 hospitalizations in a population [15–20].

The consistency of our findings with studies from other settings using more robust designs suggest that our results using the screening method provided reliable VE estimates for the Kosovo population.

Our study reflects a practical initial methodology for estimating vaccine effectiveness with routine data from available surveillance system and from vaccination coverage data. The screening method is suitable for monitoring vaccine effectiveness, and as such, it can provide crucial and timely evidence in support of public health decisions. This method could be more widely used in future pandemics, especially in low- and middle-income countries with a lack of resources for special vaccine effectiveness studies. The CIs do reflect uncertainty/randomness in the sense that the PPV and PPC estimates reflect a random ’experiment’ for people to become infected/ill or not relative to their vaccination status. The CIs, however, do not reflect uncertainty in terms of the measurements. We implicitly assume that the true values of the PPV and PCV are known, which may be debatable in the context of surveillance. The screening method employed here can be complemented with more granular study designs in order to validate the results.

Our study findings should be interpreted in light of several limitations. The unavailability of vaccine coverage statistics stratified by specific risk factors limited our ability to stratify VE estimates by demographic characteristics and vaccine types. The screening method does not allow adjustment for potential confounders. However, the objective is to quantify differences at population level. Furthermore, assuming that more vulnerable individuals were more likely to be vaccinated, the estimates may underestimate the true vaccine effectiveness for the overall population.

An essential element of preparing for future pandemic waves is developing systems that can monitor vaccine effectiveness even when relatively low resources are available. Continuous, real-time monitoring of vaccine effectiveness is crucial for effective implementation of public health measures, for informing science, and for maintaining the trust of the public [21–23]. The linkage of the communicable disease surveillance system with the vaccination coverage system during the COVID-19 pandemic, and the enrichment with detailed data including date of application of the first dose, second dose and additional doses enabled the evaluation of the vaccine effectiveness in this study.

In Kosovo, the pandemic highlighted the need to improve the quality of data in the infectious disease surveillance system and the key data and tools needed for effective vaccine monitoring programs. The digitization of health systems accelerated during the pandemic in some settings, the use of digital tools to identify and prioritize individuals for vaccination, and the linkage of vaccination data to health records with a unique identifier could improve surveillance systems in the future [24]. Before the pandemic, about 60% of low- and middle -income countries did not have electronic immunization registries [25]. This will be a surveillance priority for Kosovo going forward, and our study supports the need for enhanced surveillance systems using digitalized tools.

In conclusion, our study provides real-world evidence using routine administrative data sources to estimate COVID-19 vaccine effectiveness for Kosovo during the fourth pandemic wave when the Delta variant and later Delta plus variant were dominant. Our study using the screening method reflects an applicable initial methodology for estimating vaccine effectiveness with routine surveillance and vaccination coverage data. This method may be particularly important for low- and middle-income settings with less robust surveillance systems or fewer opportunities to conduct more robust vaccine effectiveness study designs.
